# Supplementary material for: The seminal fluid protein SFP-1 regulates mated hermaphrodite aging and fat metabolism in C. elegans
Source: EMBO J. 2025 Oct 27;44(23):7181–210. doi: 10.1038/s44318-025-00610-1 (PMC12669617; doi:10.1038/s44318-025-00610-1)
Supplement: Supplementary file 4 — Expanded View Figures [file 44318_2025_610_MOESM4_ESM.pdf]

Expanded View Figures

Figure EV1. The secreted protein SFP-1 is involved in mating-induced phenotypes, while *sfp-1* mutant male sperm and pheromones still influence hermaphrodites.

(**A**) Left panel: Diagram outlining the pathway of sperm maturation in males, beginning with mitosis and progressing through meiosis I to produce mature spermatids (pink) that are stored in the seminal vesicle (blue) prior to mating. Right panel: Fluorescence micrograph of male worms stained with MitoTracker Red (red) to visualize spermatids. SFP-1 (green) surrounds the labeled spermatids, confirming SFP-1 protein localization specifically within the seminal vesicle, as illustrated in the schematic. (Scale bars: 10 μm). (**B**) Representative fluorescence micrograph showing mating between an SFP-1::YFP-expressing male (green) and N2 hermaphrodite. The white arrow indicates the transfer of SFP-1::YFP protein (green) along with sperm (red) during copulation. The exclusive presence of sperm (red) in the uterus of N2 hermaphrodites mated with non-fluorescent control males. (Scale bars: 10 μm). (**C**) High-magnification images of the spermatheca region in hermaphrodites expressing *sfp-1::yfp*, confirming the male-specific expression of SFP-1. (Scale bars: 10 μm). (**D**) Lifespan of mated N2 worms. N2 × N2 ♂ : 10.22 ± 0.78 days, $n = 16$ worms; N2 × *sfp-1::yfp* ♂ : 8.22 ± 0.54 days, $n = 20$ worms; N2 × *sfp-1*♂ : 12.93 ± 0.93 days, $n = 22$ worms. $^{*}P = 0.025$, $^{**}P = 0.003$, indicate significance vs. the N2 × N2 ♂ group (Log-rank test). (**E**) Total brood size of N2 worms in different conditions. Fertility was assayed by measuring the total offspring production of individual hermaphrodites at 20 °C. *P* values were calculated by one-way ANOVA with Bonferroni's multiple comparisons test. $^{**}P = 0.0039$, $^{***}P = 0.0002$, $^{****}P = 4.943 \times 10^{-11}$ for comparisons against the unmated N2 control; $^{****}P = 5.02 \times 10^{-7}$ for N2 × N2 ♂ vs. N2 × *sfp-1*♂. Data are presented as mean ± SEM, based on at least 15 worms for each condition. (**F**) Body length of N2 worms was measured under different mating and genetic conditions over seven days, with statistical analysis performed separately for each day using ordinary one-way ANOVA followed by Bonferroni's multiple comparisons test against the N2 unmated control group. On day 3, *gonEx17*: $^{***}P = 1.66 \times 10^{-4}$; N2 × N2 ♂: $^{****}P = 2.02 \times 10^{-6}$; N2 × *sfp-1*♂: $^{****}P = 8.35 \times 10^{-8}$. On day 4, *gonEx17*: $^{*}P = 0.014$; N2 × N2 ♂: $^{***}P = 4.11 \times 10^{-4}$; N2 × *sfp-1*♂: $^{****}P = 1.24 \times 10^{-6}$. On day 5, *gonEx17*: $^{****}P = 1.42 \times 10^{-7}$; N2 × N2 ♂: $^{****}P = 2.24 \times 10^{-7}$; N2 × *sfp-1*♂: $^{****}P = 1.44 \times 10^{-10}$. On day 6, *gonEx17*: $^{****}P = 2.06 \times 10^{-9}$; N2 × N2 ♂: $^{****}P = 3.87 \times 10^{-9}$; N2 × *sfp-1*♂: $^{****}P = 4.09 \times 10^{-12}$. On day 7, *gonEx17*: $^{****}P = 1.09 \times 10^{-12}$; N2 × N2 ♂: $^{****}P = 8.00 \times 10^{-15}$; N2 × *sfp-1*♂: $^{****}P = 2.50 \times 10^{-14}$. Data are presented as mean ± SEM, based on at least 15 worms for each condition. (**G**) Lifespan of N2 under different male conditions. N2 in no male condition: 16.96 ± 0.76 days, $n = 36$ worms; N2 in N2 male condition: 11.72 ± 0.28 days, $n = 97$ worms; N2 in *sfp-1* male condition: 10.89 ± 0.31 days, $n = 79$ worms, $P = 0.066$; $^{****}P = 9.52 \times 10^{-12}$, comparisons were made using the Log-rank (Mantel–Cox) test.

 © The Author(s)

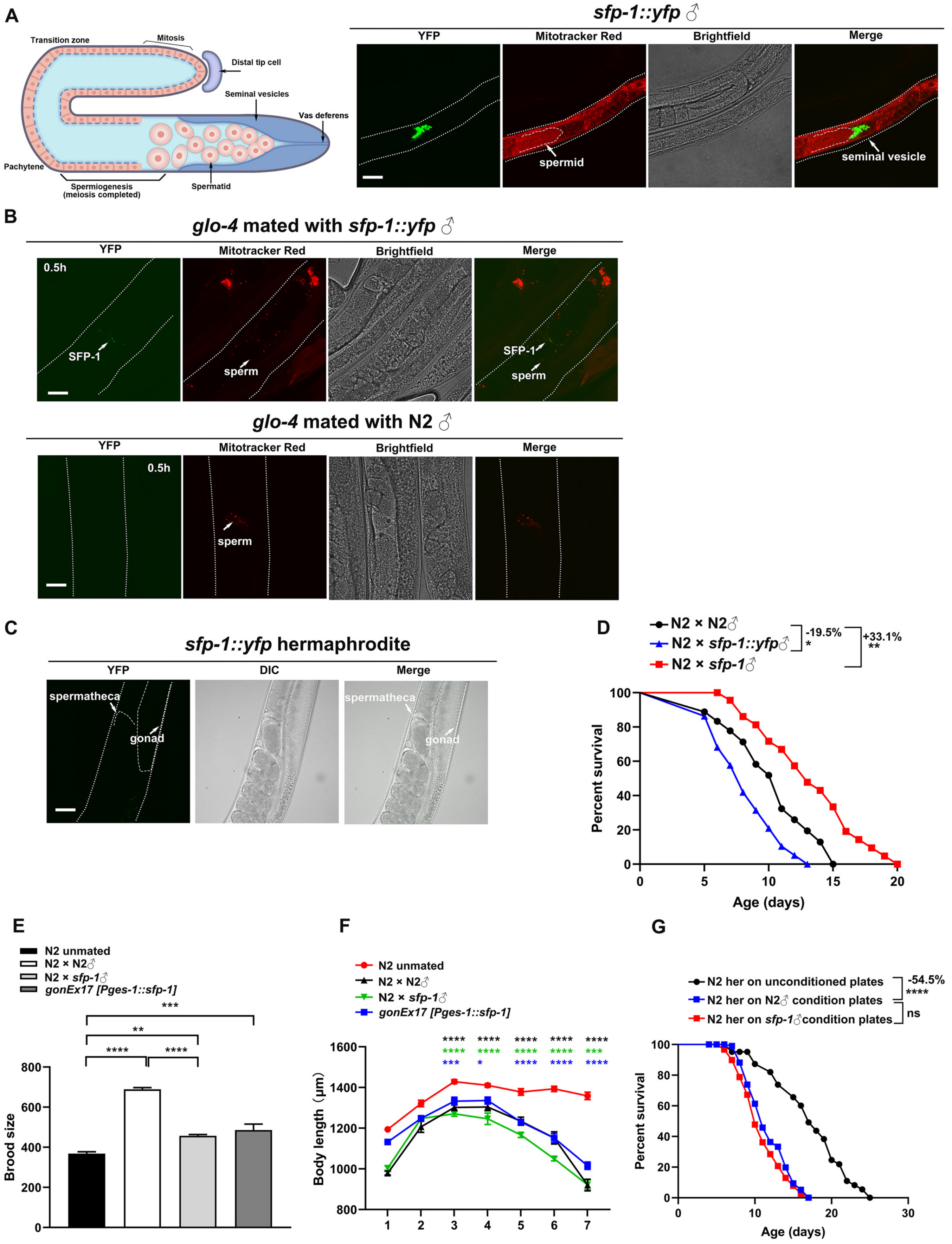
A
sfp-1::yfp ♂
Transition zone
Mitosis
Distal tip cell
Seminal vesicles
Vas deferens
Pachytene
Spermiogenesis (meiosis completed)
Spermatid
YFP
Mitotracker Red
Brightfield
Merge
spermid
seminal vesicle
B
glo-4 mated with sfp-1::yfp ♂
0.5h
SFP-1
sperm
glo-4 mated with N2 ♂
C
sfp-1::yfp hermaphrodite
DIC
spermatheca
gonad
D
N2 × N2♂
N2 × sfp-1::yfp♂
N2 × sfp-1♂
-19.5%
+33.1%
Percent survival
Age (days)
E
N2 unmated
N2 × N2♂
N2 × sfp-1♂
gonEx17 [Pges-1::sfp-1]
Brood size
F
Body length（μm）
G
N2 her on unconditioned plates
N2 her on N2♂ condition plates
N2 her on sfp-1♂condition plates
-54.5%
ns

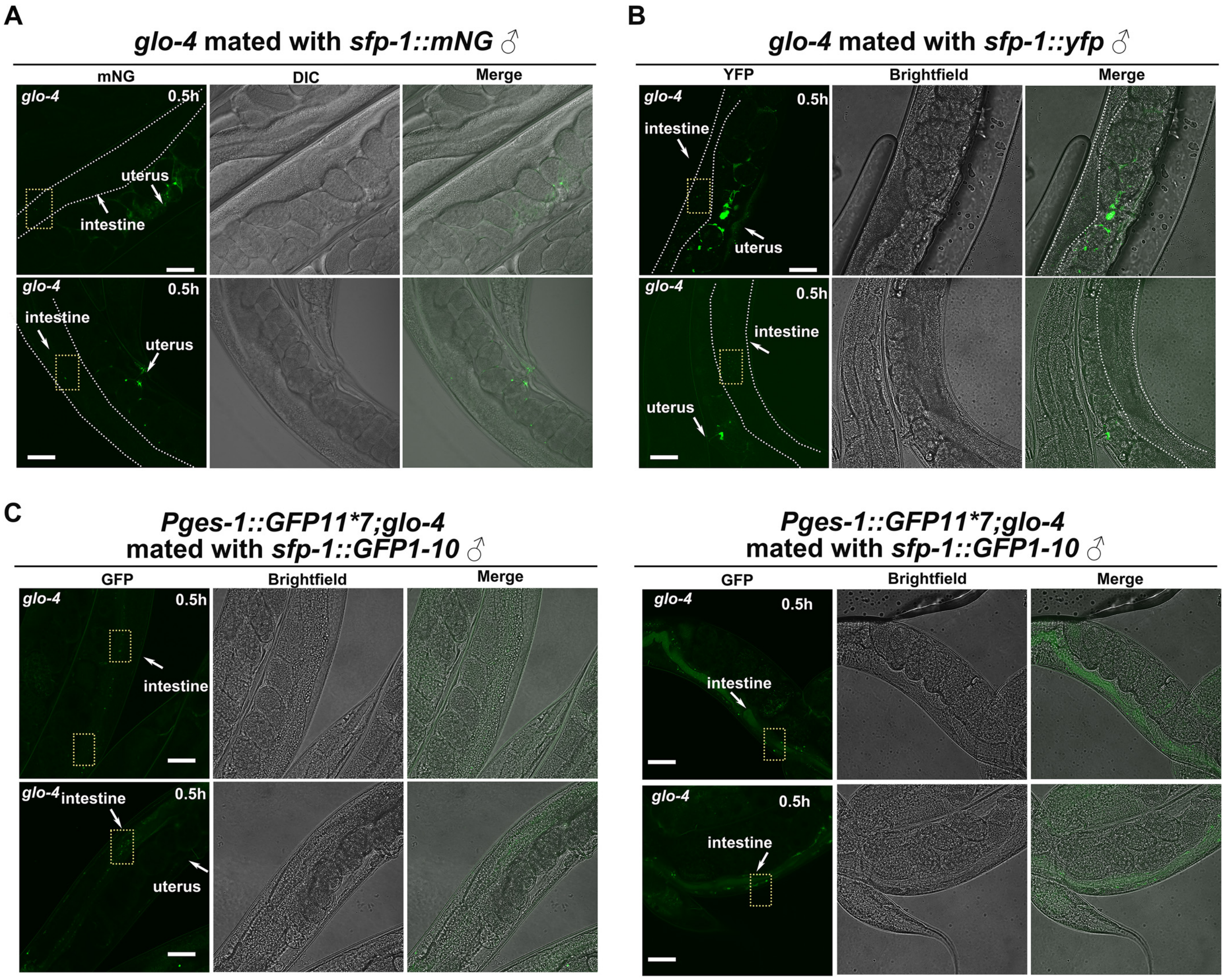

Figure EV2. SFP-1 is transferred from the uterus into the intestinal cells in mated hermaphrodites.

(**A**) Male-to-hermaphrodite transfer of SFP-1::YFP. *sfp-1::yfp* males were mated with *glo-4* hermaphrodites (low gut autofluorescence). Imaging of mated *glo-4* hermaphrodites revealed the presence of male-derived SFP-1::YFP in the uterus immediately after copulation. Within 30 min post-mating, weak fluorescence signals were detected in intestinal cells. The yellow rectangle highlights the fluorescent signal observed in the intestine. (Scale bars: 10 μm). (**B**) Male-to-hermaphrodite transfer of SFP-1::YFP. *sfp-1::yfp* males were mated with *glo-4* hermaphrodites (low gut autofluorescence). Imaging of mated *glo-4* hermaphrodites revealed the presence of male-derived SFP-1::YFP in the uterus immediately after copulation. Within 30 min post-mating, weak fluorescence signals were detected in intestinal cells. The yellow rectangle highlights the fluorescent signal observed in the intestine. (Scale bars: 10 μm). (**C**) Male-to-hermaphrodite transfer of SFP-1::GFP1-10. *sfp-1::GFP1-10* males were mated with *Pges-1::GFP11*7;glo-4* hermaphrodites. Half an hour after mating, bright fluorescence appeared in the intestine cell, confirming the transfer and endocytosis of SFP-1. Autofluorescence from male sperm was observed. The yellow rectangle highlights the fluorescent signal observed in the intestine. (Scale bars: 10 μm).

 © The Author(s)

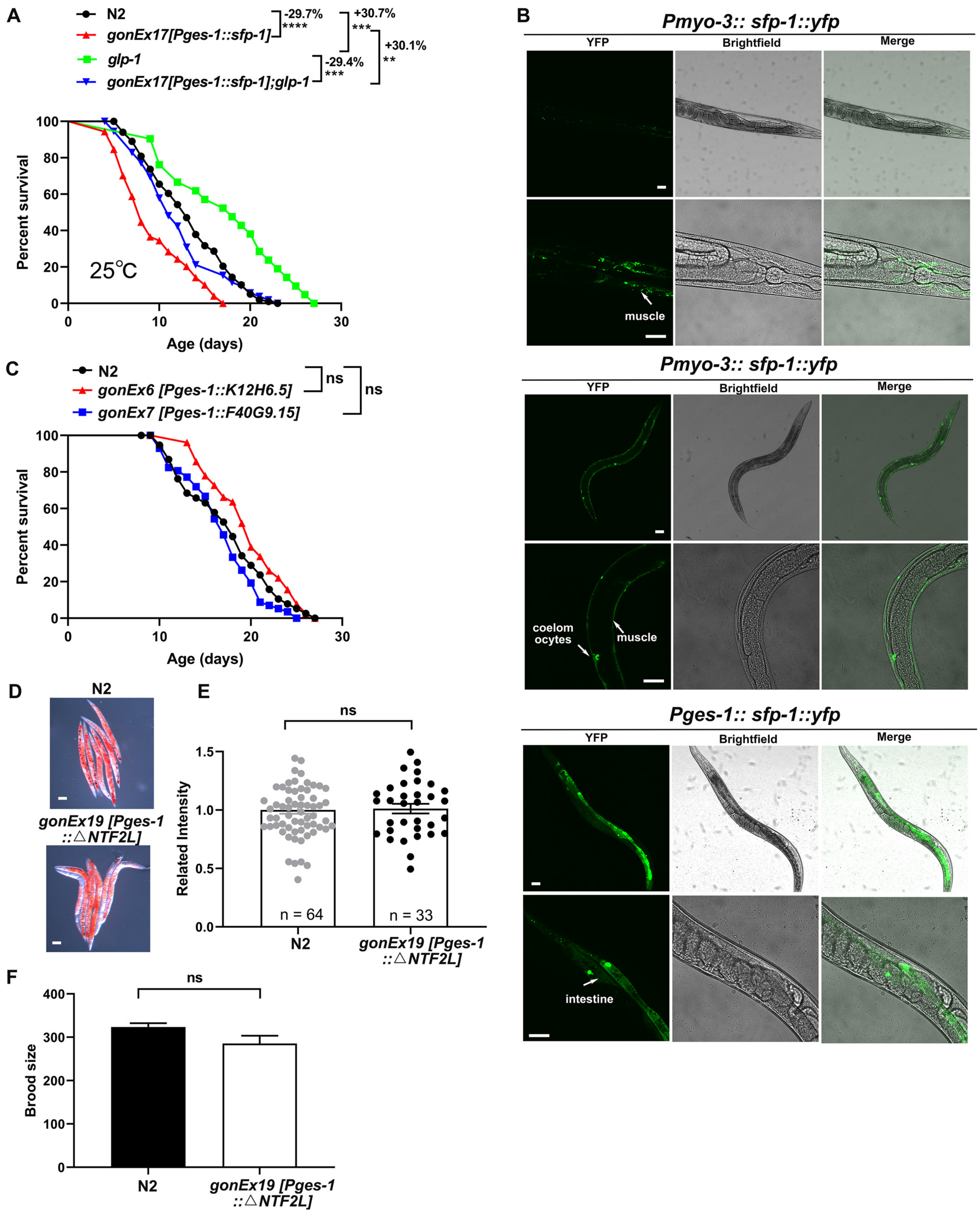
A
N2
gonEx17[Pges-1::sfp-1]
glp-1
gonEx17[Pges-1::sfp-1];glp-1
-29.7%
****
+30.7%
***
+30.1%
**
-29.4%
***
Percent survival
25°C
Age (days)
B
Pmyo-3:: sfp-1::yfp
YFP
Brightfield
Merge
muscle
Pmyo-3:: sfp-1::yfp
coelom ocytes
muscle
Pges-1:: sfp-1::yfp
intestine
C
N2
gonEx6 [Pges-1::K12H6.5]
gonEx7 [Pges-1::F40G9.15]
ns
ns
Percent survival
Age (days)
D
N2
gonEx19 [Pges-1 ::△NTF2L]
E
ns
Related Intensity
n = 64
n = 33
N2
gonEx19 [Pges-1 ::△NTF2L]
F
ns
Brood size
N2
gonEx19 [Pges-1 ::△NTF2L]

Figure EV3. The potential function of NTF2L in longevity, lipid metabolism, and reproduction.

(**A**) Lifespan survival curves of *glp-1(e2141)* worms, *gonEx17* worms, wild-type N2 worms and *gonEx17;glp-1(e2141)* worms when grown and aged at 25 °C. N2: 13.17 ± 0.46 days, $n = 98$ worms; *gonEx17 [Pges-1::sfp-1]*: 9.25 ± 0.52, $n = 53$ worms; *glp-1(e2141)*: 17.33 ± 1.30, $n = 21$ worms; *gonEx17 [Pges-1::sfp-1];glp-1(e2141)*: 12.10 ± 0.63, $n = 52$ worms. Comparisons were made using the Log-rank (Mantel–Cox) test, $^{****}P = 5.44 \times 10^{-8}$, $^{***}P = 0.0003$; $^{***}P = 0.00015$; $^{**}P = 0.01$ (from left to right). (**B**) Expression patterns of *Pmyo-3::sfp-1::yfp* and *Pges-1::sfp-1::yfp*. In *Pmyo-3::sfp-1::yfp* animals, low-magnification images show whole worms, while high-magnification images highlight coelomocytes and muscles. Bright fluorescent signals were observed in coelomocytes. In *Pges-1::sfp-1::yfp* animals, low-magnification images show whole worms, and high-magnification images focus on the intestine. Scale bars: 50 µm (low magnification), 10 µm (high magnification). (**C**) Lifespan survival curves of WT animals and intestinal overexpressing K12H6.5 or F40G9.15 animals. The other seminal fluid proteins such as K12H6.5 and F40G9.15 in the intestine had almost no effect on longevity. N2: 17.40 ± 0.79 days, $n = 48$ worms; *gonEx6 [Pges-1::K12H6.5]*: 19.61 ± 0.46 days, $n = 77$ worms; *gonEx7 [Pges-1::F40G9.15]*: 16.74 ± 0.53 days, $n = 57$ worms, comparisons were made using the Log-rank (Mantel–Cox) test. (**D, E**) Representative pictures of Oil Red O staining in day 5 adult control and *gonEx19 [Pges-1::△NTF2]* worms. Quantification of Oil Red O fat staining. Compared to the control, the neutral lipid level difference was abolished in the absence of NTFL. Data are presented as mean ± SEM, based on at least 32 worms for each condition. *P* values were calculated by two-tailed unpaired *t* test. (**F**) Total brood size of N2 worms and *gonEx19 [Pges-1::△NTF2]* worms. In contrast to the fertility-promoting effects observed in mated N2 and *intestine::sfp-1* transgenic worms, the lack of NTF2L significantly inhibited fertility. Data are presented as mean ± SEM, based on at least 8 worms for each condition. *P* values were calculated by two-tailed unpaired *t* test.

© The Author(s)

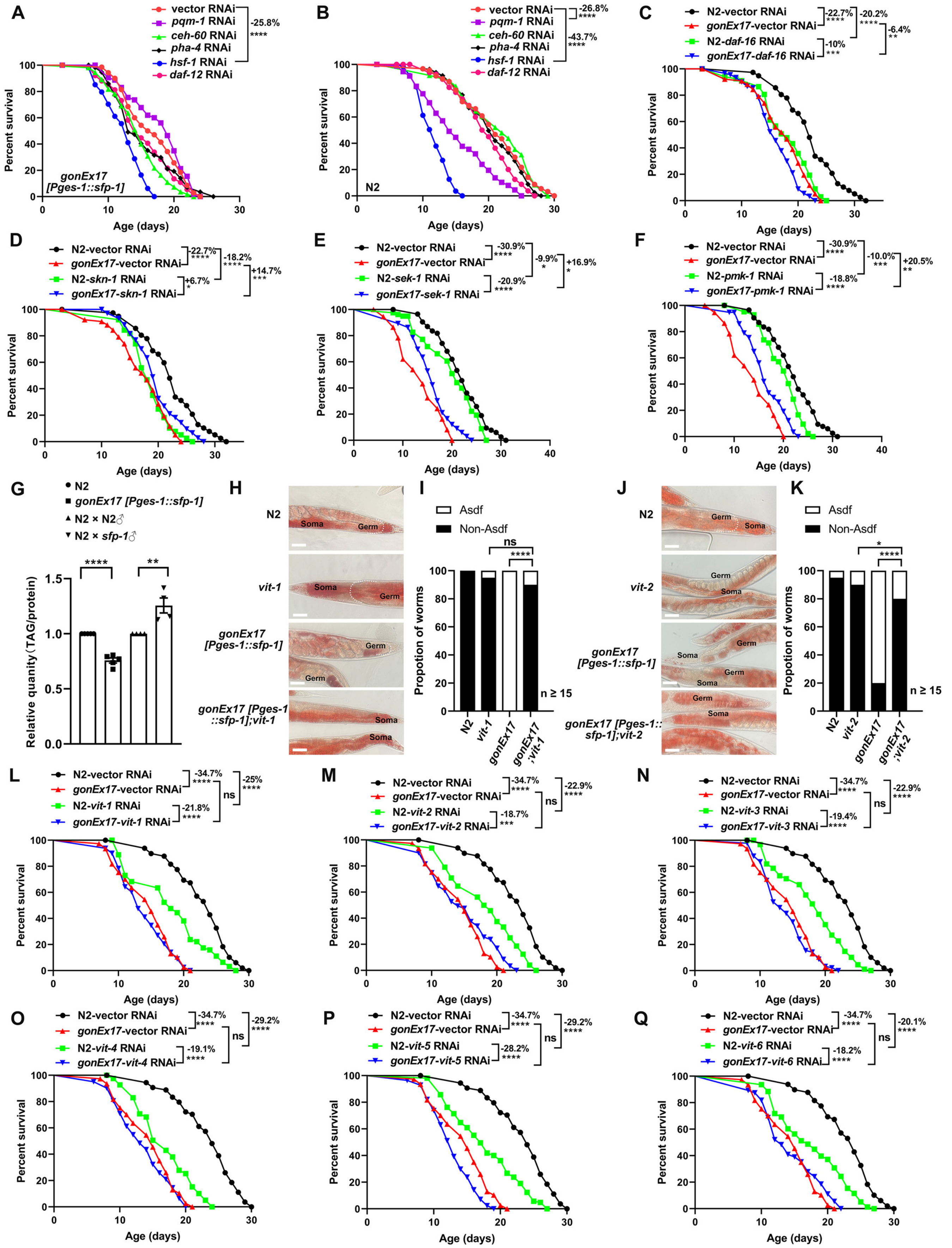
A
B
C
D
E
F
G
H
I
J
K
L
M
N
O
P
Q
vector RNAi
pqm-1 RNAi
ceh-60 RNAi
pha-4 RNAi
hsf-1 RNAi
daf-12 RNAi
gonEx17 [Pges-1::sfp-1]
N2
N2-vector RNAi
gonEx17-vector RNAi
N2-daf-16 RNAi
gonEx17-daf-16 RNAi
N2-skn-1 RNAi
gonEx17-skn-1 RNAi
N2-sek-1 RNAi
gonEx17-sek-1 RNAi
N2-pmk-1 RNAi
gonEx17-pmk-1 RNAi
Percent survival
Age (days)
N2 × N2♂
N2 × sfp-1♂
Relative quanity (TAG/protein)
Soma
Germ
vit-1
vit-2
gonEx17 [Pges-1::sfp-1];vit-1
gonEx17 [Pges-1::sfp-1];vit-2
Asdf
Non-Asdf
Propotion of worms
n ≥ 15
N2-vit-1 RNAi
gonEx17-vit-1 RNAi
N2-vit-2 RNAi
gonEx17-vit-2 RNAi
N2-vit-3 RNAi
gonEx17-vit-3 RNAi
N2-vit-4 RNAi
gonEx17-vit-4 RNAi
N2-vit-5 RNAi
gonEx17-vit-5 RNAi
N2-vit-6 RNAi
gonEx17-vit-6 RNAi
ns

Figure EV4. Knockdown of the SKN-1 signal pathway major involving genes increased the lifespan of intestinal overexpressing SFP-1 transgenic line.

(**A**) Lifespan survival curves of intestinal SFP-1-overexpressing animals treated with RNAi targeting classical longevity-associated transcription factors. None of the tested transcription factors extended the lifespan of intestinal SFP-1-overexpressing animals. *gonEx17-vector* RNAi: 16.76 ± 0.51 days (*n* = 70); *gonEx17-pqm-1* RNAi: 17.77 ± 0.80 days (*n* = 52); *gonEx17-ceh-60* RNAi: 14.58 ± 0.52 days (*n* = 55); *gonEx17-pha-4* RNAi: 15.33 ± 0.62 days (*n* = 57); *gonEx17-hsf-1* RNAi:12.43 ± 0.40 days (*n* = 51); *gonEx17-daf-12* RNAi: 15.26 ± 0.52 days (*n* = 74). $^{****}P = 2.19 \times 10^{-9}$. Significance was determined by the Log-rank (Mantel–Cox) test. (**B**) Lifespan survival curves of WT (N2) animals treated with classical transcription factor genes RNAi. N2-vector RNAi: 20.71 ± 0.55 days (*n* = 92); N2-*pqm-1* RNAi:15.16 ± 0.56 days (*n* = 88); N2-*ceh-60* RNAi: 21.19 ± 0.79 days (*n* = 48); N2-*pha-4* RNAi: 20.36 ± 0.53 days (*n* = 80); N2-*hsf-1* RNAi: 11.66 ± 0.31 days (*n* = 56); N2-*daf-12* RNAi: 19.44 ± 0.49 days (*n* = 80). $^{****}P = 3.64 \times 10^{-11}$ (N2-vector RNAi vs. N2-*pqm-1* RNAi). $^{****}P = 6.20 \times 10^{-12}$ (N2-vector RNAi vs. N2-*hsf-1* RNAi). Significance was determined by the Log-rank (Mantel–Cox) test. (**C**) Knockdown of transcription factor gene *daf-16* by RNAi reduces the lifespan of SFP-1-overexpressing worms. N2-vector RNAi: 22.21 ± 0.55 days (*n* = 77); N2-*daf-16* RNAi:17.76 ± 0.58 days (*n* = 67); *gonEx17*-vector RNAi: 17.06 ± 0.53 days (*n* = 76); *gonEx17-daf-16* RNAi: 15.95 ± 0.40 days (*n* = 90). $^{****}P = 1.10 \times 10^{-10}$ (N2-vector RNAi vs. *gonEx17*-vector RNAi), $^{***}P = 1.58 \times 10^{-4}$ (N2-*daf-16* RNAi vs. *gonEx17-daf-16* RNAi), $^{****}P = 4.69 \times 10^{-8}$ (N2-*daf-16* RNAi vs. N2-vector RNAi), $^{**}P = 0.003$. Significance was determined by the Log-rank (Mantel–Cox) test. (**D**) Knockdown of transcription factor gene *skn-1* by RNAi extends the lifespan of SFP-1-overexpressing worms. N2-vector RNAi: 22.21 ± 0.55 days (*n* = 77); N2-*skn-1* RNAi:18.39 ± 0.37 days (*n* = 77); *gonEx17*-vector RNAi: 17.06 ± 0.53 days (*n* = 76); *gonEx17-skn-1* RNAi: 19.50 ± 0.40 days (*n* = 104). $^{****}P = 1.10 \times 10^{-10}$ (N2-vector RNAi vs. *gonEx17*-vector RNAi), $^{****}P = 1.80 \times 10^{-9}$ (N2-*skn-1* RNAi vs. N2-vector RNAi), $^{***}P = 0.001$, $^{*}P = 0.012$. Significance was determined by the Log-rank (Mantel–Cox) test. (**E**, **F**) Knockdown of SKN-1 upstream effector *sek-1* (**E**) and *pmk-1* (**F**) by RNAi extends the lifespan of SFP-1 expressed ectopically worms. N2-vector RNAi: 22.00 ± 0.43 days (*n* = 116); N2-*sek-1* RNAi:19.81 ± 0.64 days (*n* = 71); *gonEx17*-vector RNAi: 13.43 ± 0.71 days (*n* = 37); *gonEx17-sek-1* RNAi: 15.71 ± 0.48 days (*n* = 66); N2-*pmk-1* RNAi: 19.79 ± 0.58 days (*n* = 42); *gonEx17-pmk-1* RNAi: 16.18 ± 0.52 days (*n* = 57). For (**E**), $^{****}P = 4.97 \times 10^{-22}$ (N2-vector RNAi vs. *gonEx17*-vector RNAi), $^{****}P = 1.17 \times 10^{-8}$ (N2-*sek-1* RNAi vs. *gonEx17-sek-1* RNAi), $^{*}P = 0.021$, $^{*}P = 0.026$. For (**F**), $^{****}P = 4.97 \times 10^{-22}$ (N2-vector RNAi vs. *gonEx17*-vector RNAi), $^{****}P = 1.07 \times 10^{-5}$ (N2-*pmk-1* RNAi vs. *gonEx17-pmk-1* RNAi), $^{***}P = 0.001$, $^{**}P = 0.003$. Significance was determined by the Log-rank (Mantel–Cox) test. (**G**) Comparative analysis of triglyceride (TAG) levels in N2 worms under different mating conditions. Data are presented as mean ± SEM from ≥4 independent biological replicates. Statistical significance was determined by two-tailed unpaired Student's *t* test: $^{****}P = 5.51 \times 10^{-6}$ (N2 vs. *gonEx17*); $^{**}P = 0.0095$ (N2 × N2 vs. N2 × *sfp-1*). Error bars represent SEM. Note: Each mated condition was normalized to its respective control group (N2 or N2 × N2) due to the substantial post-mating TAG reduction. (**H**, **I**) The absence of *vit-1* suppressed somatic lipid depletion (Asdf). Lipid distribution was assessed in at least 15 animals. $^{****}P < 1 \times 10^{-15}$ and *P* values were obtained Chi-square. (Scale bars: 10 μm). (**J**, **K**) The absence of *vit-2* suppressed somatic lipid depletion (Asdf). Lipid distribution was assessed in at least 15 animals. $^{****}P < 1 \times 10^{-15}$, $^{*}P = 0.047$, and *P* values were obtained by Chi-square. (Scale bars: 10 μm). (**L–Q**) Lifespan survival curves of WT animals and intestinal overexpressing SFP-1 animals treated with EV or *vit* RNAi. *vit* RNAi had almost no effect on the lifespan of SFP-1 expressed ectopically worms. N2-vector RNAi: 22.79 ± 0.61 days (*n* = 49); *gonEx17*-vector RNAi: 15.33 ± 0.42 days (*n* = 67); N2-*vit-1* RNAi: 17.81 ± 0.71 days (*n* = 63); *gonEx17-vit-1* RNAi: 13.84 ± 0.42 days (*n* = 78); N2-*vit-2* RNAi: 18.15 ± 0.73days (*n* = 48); *gonEx17-vit-2* RNAi: 14.72 ± 0.62 days (*n* = 59); N2-*vit-3* RNAi: 18.06 ± 0.53 days (*n* = 88); *gonEx17-vit-3* RNAi: 13.89 ± 0.38 days (*n* = 91); N2-*vit-4* RNAi: 16.71 ± 0.66 days (*n* = 40); *gonEx17-vit-4* RNAi: 13.40 ± 0.45 days (*n* = 62); N2-*vit-5* RNAi: 17.70 ± 0.51 days (*n* = 105); *gonEx17-vit-5* RNAi: 12.73 ± 0.35 days (*n* = 84); N2-*vit-6* RNAi: 17.50 ± 0.59 days (*n* = 78); *gonEx17-vit-6* RNAi: 14.31 ± 0.59 days (*n* = 54). $^{****}P = 5.49 \times 10^{-19}$ (N2-vector RNAi vs. *gonEx17*-vector RNAi). For (**L**), $^{****}P = 3.50 \times 10^{-8}$ (N2-*vit-1* RNAi vs. *gonEx17-vit-1* RNAi), $^{****}P = 4.90 \times 10^{-5}$ (N2-*vit-1* RNAi vs. N2-vector RNAi). For (**M**), $^{***}P = 1.19 \times 10^{-4}$ (N2-*vit-2* RNAi vs. *gonEx17-vit-2* RNAi), $^{****}P = 2.89 \times 10^{-6}$ (N2-*vit-2* RNAi vs. N2-vector RNAi). For (**N**), $^{****}P = 2.53 \times 10^{-11}$ (N2-*vit-3* RNAi vs. *gonEx17-vit-3* RNAi), $^{****}P = 8.26 \times 10^{-8}$ (N2-*vit-3* RNAi vs. N2-vector RNAi). For (**O**), $^{***}P = 8.00 \times 10^{-5}$ (N2-*vit-4* RNAi vs. *gonEx17-vit-4* RNAi), $^{****}P = 3.13 \times 10^{-10}$ (N2-*vit-4* RNAi vs. N2-vector RNAi). For (**P**), $^{****}P = 1.95 \times 10^{-14}$ (N2-*vit-5* RNAi vs. *gonEx17-vit-5* RNAi), $^{****}P = 3.50 \times 10^{-7}$ (N2-*vit-5* RNAi vs. N2-vector RNAi). For (**Q**), $^{****}P = 2.30 \times 10^{-5}$ (N2-*vit-6* RNAi vs. *gonEx17-vit-6* RNAi), $^{****}P = 1.42 \times 10^{-7}$ (N2-*vit-6* RNAi vs. N2-vector RNAi). Significance was determined by the Log-rank (Mantel–Cox) test.

 © The Author(s)

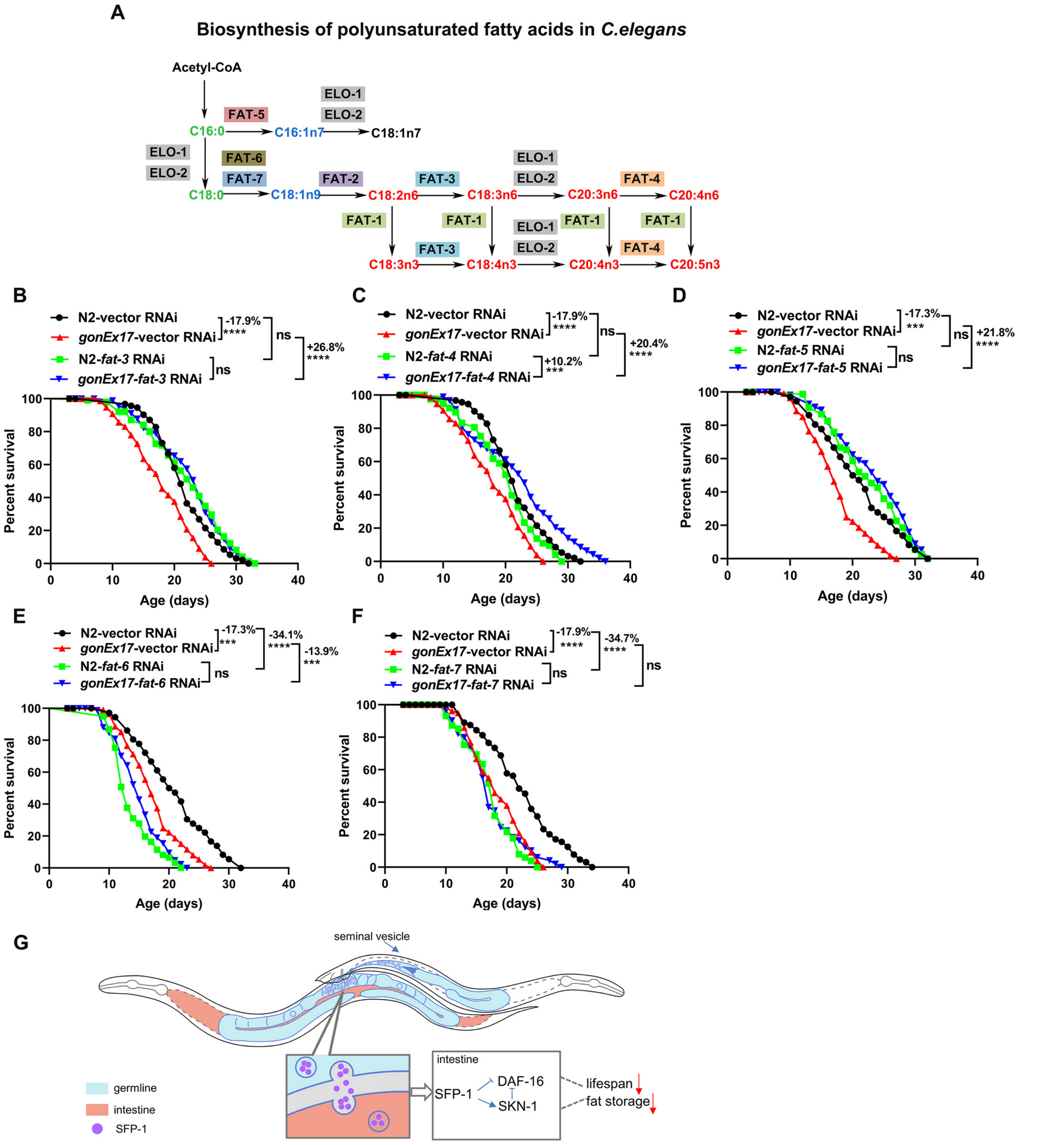
A
Biosynthesis of polyunsaturated fatty acids in C.elegans
Acetyl-CoA
FAT-5
ELO-1
ELO-2
C16:0
C16:1n7
C18:1n7
FAT-6
FAT-7
C18:0
C18:1n9
FAT-2
C18:2n6
FAT-3
C18:3n6
C20:3n6
FAT-4
C20:4n6
FAT-1
C18:3n3
C18:4n3
C20:4n3
C20:5n3
B
N2-vector RNAi
gonEx17-vector RNAi
N2-fat-3 RNAi
gonEx17-fat-3 RNAi
-17.9%
****
ns
+26.8%
Percent survival
Age (days)
C
N2-fat-4 RNAi
gonEx17-fat-4 RNAi
+10.2%
***
+20.4%
D
N2-fat-5 RNAi
gonEx17-fat-5 RNAi
-17.3%
+21.8%
E
N2-fat-6 RNAi
gonEx17-fat-6 RNAi
-34.1%
-13.9%
F
N2-fat-7 RNAi
gonEx17-fat-7 RNAi
-34.7%
G
seminal vesicle
germline
intestine
SFP-1
SFP-1
DAF-16
SKN-1
lifespan
fat storage

Figure EV5. Fatty acid desaturases are involved in the lifespan regulation of intestinal overexpressing SFP-1 animals.

(**A**) The pathway of PUFA synthesis in *Caenorhabditis elegans*. (**B**, **C**) Lifespan survival curves of WT animals and intestinal SFP-1 overexpressing (*intestine::sfp-1,gonEx17*) animals treated with EV or *fat* RNAi. Knockdown of *fat-3* (**B**) and *fat-4* (**C**) suppressed the short-lived phenotype of *intestine::sfp-1* transgenic worms. (**B**) N2-vector RNAi: 22.41 ± 0.75 days ($n = 64$); *gonEx17*-vector RNAi: 18.39 ± 0.49 days ($n = 77$); N2-*fat-3* RNAi: 23.96 ± 0.52 days ($n = 138$); *gonEx17-fat-3* RNAi: 23.32 ± 0.62 days ($n = 98$). $^{****}P = 1.13 \times 10^{-6}$, $^{****}P = 2.06 \times 10^{-11}$ (from left to right). (**C**) N2-vector RNAi: 22.41 ± 0.75 days (n = 64); *gonEx17*-vector RNAi: 18.39 ± 0.49 days ($n = 77$); N2-*fat-4* RNAi: 19.91 ± 0.60 days ($n = 74$); *gonEx17-fat-4* RNAi: 22.15 ± 0.75 days ($n = 93$). $^{****}P = 1.13 \times 10^{-6}$, $^{***}P = 0.0007$; $^{****}P = 5.17 \times 10^{-7}$ (from left to right). (**D**) Lifespan survival curves of WT animals and intestinal SFP-1 overexpressing (*intestine::sfp-1,gonEx17*) animals treated with EV or *fat-5* RNAi. Knockdown of *fat-5* suppressed the short-lived phenotype of *intestine::sfp-1* transgenic worms. N2-vector RNAi: 20.81 ± 1.01 days (n = 36); *gonEx17*-vector RNAi: 17.29 ± 0.41 days ($n = 113$); N2-*fat-5* RNAi: 21.31 ± 0.58 days ($n = 84$); *gonEx17-fat-5* RNAi: 21.06 ± 0.94 days ($n = 48$). $^{***}P = 1.07 \times 10^{-4}$, $^{****}P = 1.58 \times 10^{-6}$. (**E**, **F**) Lifespan survival curves of WT animals and *intestine::sfp-1* animals treated with EV or *fat* RNAi. *fat-6* (**E**) and *fat-7* (**F**) RNAi had almost no effect on the lifespan of worms with ectopic SFP-1 expression. (**E**) N2-vector RNAi: 20.81 ± 1.01 days ($n = 36$); N2-*fat-6* RNAi: 13.71 ± 0.43 days ($n = 61$); *gonEx17*-vector RNAi: 17.29 ± 0.41 days ($n = 113$); *gonEx17-fat-6* RNAi: 14.89 ± 0.42 days ($n = 84$). $^{***}P = 1.07 \times 10^{-4}$, $^{****}P = 4.04 \times 10^{-10}$, $^{***}P = 5.18 \times 10^{-5}$ (from left to right). (**F**) N2-vector RNAi: 22.41 ± 0.75 days ($n = 64$); N2-*fat-7* RNAi: 17.25 ± 0.56 days ($n = 51$); *gonEx17*-vector RNAi: 18.39 ± 0.49 days ($n = 77$); *gonEx17-fat-7* RNAi: 17.50 ± 0.66 days ($n = 49$). $^{****}P = 1.13 \times 10^{-6}$, $^{****}P = 2.45 \times 10^{-8}$ (from left to right). (**G**) A schematic model illustrating the pathway of SFP-1 transport and the regulation pathways in post-mating longevity and fat metabolism.

© The Author(s)
